# Genome Sequences of Three Spore-Forming Bacteria Isolated from the Feces of Organically Raised Chickens

**DOI:** 10.1128/genomeA.00880-16

**Published:** 2016-09-01

**Authors:** Victoria Kennedy, Tricia A. Van Laar, Omoshola Aleru, Michael Thomas, Michelle Ganci, Mamta Rawat

**Affiliations:** a Department of Animal Science, California State University, Fresno, Fresno, California, USA; b Department of Biology, California State University, Fresno, Fresno, California, USA

## Abstract

Antibiotic feed supplements have been implicated in the rise of multidrug-resistant bacteria. An alternative to antibiotics is probiotics. Here, we report the genome sequences of two *Bacillus* and one *Solibacillus* species, all spore-forming, Gram-positive bacteria, isolated from the feces organically raised chicken feces, with potential to serve as probiotics.

## GENOME ANNOUNCEMENT

Antibiotic feed supplements have been used in commercial poultry farming for a number of years to promote growth and prevent disease. However, the spread of antibiotic resistance has been linked to the widespread use of these antibiotics, in particular resistance to fluoroquinolones (1). In 2006, the European Union (EU) banned antibiotics in chicken feed and in 2015 (2), the United States Food and Drug Administration issued a Veterinary Feed Directive restricting the use of antibiotics only for assuring animal health under veterinary supervision (http://www.fda.gov). As a result, there is great interest in alternative growth-promoting and prophylactic feed supplements, such as probiotics, live microorganisms that confer a health benefit on the host. Many probiotic products are already available for commercially farmed poultry, and a number of these contain bacterial spores of the genus *Bacillus* (3). Bacterial spores are well suited for use as probiotics because they are metabolically dormant and resistant to environmental stresses resulting in long shelf life during distribution and storage (4). Furthermore, they are able to survive the bactericidal environment of the stomach and germinate in the chicken gastrointestinal tract where they become metabolically active, secreting either antimicrobial compounds and/or competing for essential nutrients with the normal microbiome (5).

Here, we report the genome sequences of three strains: *Bacillus safensis* MROC1*, Bacillus cereus* MROC2*,* and *Solibacillus silvestris* MROC3, a *Bacillus*-like organism, isolated from the feces of organically raised chickens. After enrichment and isolation, axenic cultures of each strain were grown in Trypticase soy broth. The cells were harvested by centrifugation at 15,000 rpm for 10 min. DNA was isolated using the Wizard Genomic DNA purification kit (Promega) using the protocol for Gram-positive bacterium. The genomic DNA was sent to MRDNA (Shallowater, TX) for library preparation and sequenced on an Illumina MiSeq generating 1 to 2 million 300 bp paired-end reads with 50 to 100× coverage. Assembly of the genomes was performed using the A5-MiSeq assembly pipeline (6). The genome of *B. safensis* was assembled into 18 contigs, with a total length of 3,618,371 bp (*N*_50_, 876,618 bp) and 42.1% G+C content. The genome of *B. cereus* was assembled into 40 contigs with a total length of 5,458,745 bp (*N*_50_, 508,547 bp) and 35.0% G+C content. The genome of *S. silvestris* was assembled into 139 contigs with a total length of 4,188,698 bp (*N*_50_, 390,099 bp) and 38.5% G+C content. The genomes were annotated and mapped to the closest neighbor using RAST (7). Genome mining of these three strains may be able to provide insight into the potential of these strains as probiotics.

### Accession number(s).

The whole-genome shotgun project sequences for *B. safensis* MROC1, *B. cereus* MROC2, and *S. silvestris* MROC3 have been deposited at DDBJ/ENA/GenBank, under the accession numbers LZRH00000000, LZRI00000000, and LZRJ00000000, respectively. The versions described in this paper are LZRH01000000 (MROC1), LZRI01000000 (MROC2), and LZRJ01000000 (MROC3).
